# Glioblastoma: two immune subtypes under the surface of the cold tumor

**DOI:** 10.18632/aging.204067

**Published:** 2022-05-23

**Authors:** Wu Xiong, Cong Li, Guang Kong, Bowen Wan, Siming Wang, Jin Fan

**Affiliations:** 1 Department of Orthopedics, The First Affiliated Hospital of Nanjing Medical University, Nanjing, Jiangsu, China; 2 Nanjing Medical University, Nanjing, Jiangsu, China; 3 Department of Orthopedics, Clinical Medical College of Yangzhou University, Subei People's Hospital of Jiangsu, Yangzhou, Jiangsu, China

**Keywords:** immunity, glioblastoma, tumor microenvironment, immunotherapy, biomarker

## Abstract

Glioblastoma is classified as an immunocompromised tumor. The immune pattern beneath the cold tumor surface, however, has yet to be confirmed. Understanding the immune pattern of glioblastoma will aid in the development of effective treatment strategies. We performed weighted gene co-expression network analysis on all immune-related genes in TCGA-GBM transcriptional data and screened 35 prognosis-related immune genes. Unsupervised consistent clustering of these genes was used to analyze the immunological pattern of GBM. A glioblastoma immune prognostic score was developed by using 13 genes discovered by cox regression methods and verified with the GEO dataset to assess the immune profile, prognosis, and immunotherapy effects in individual patients. Glioblastoma has two immune modalities, immune tolerance and immunodeficiency, with distinct immune microenvironments, tumor-associated macrophages being one of the most promising new therapeutic targets. GIPS is a promising biomarker for assessing immune evasion mechanisms, immunotherapy responses, and prognosis in patients.

## INTRODUCTION

Glioblastoma is a highly malignant tumor of the central nervous system [1], and patients’ prognoses remain dismal despite the availability of numerous treatments such as surgery, radiotherapy, and chemotherapy [2, 3]. Recently, immunotherapy has become increasingly popular, with immune checkpoint inhibitors serving as a new anti-tumor treatment [4, 5]. Immune system function can be suppressed to prevent autoimmune illnesses via immune checkpoints, but if they are suppressed too much, the body's ability to identify and eliminate aberrant cells is compromised [6]. Inhibitors of immunological checkpoints have anti-tumor effects through inhibiting the immune checkpoints' function, but their success in patients with glioblastoma has been uneven. A major cause of immunotherapy's poor efficacy is a lack of understanding of the immunological characteristics of glioblastoma. Individualized immunotherapy for glioblastoma can be achieved by analyzing each patient's immunological profile. However, glioblastoma’s immune milieu is poorly understood. It’s critical to clarify the immune trait of glioblastoma and develop marks that can accurately assess the immunological profile and prognosis of patients.

The goal of this study was to determine the immunological pattern of glioblastoma and create a marker that may be used to assess the prognosis and immune profile of GBM patients. WGCNA analysis of all immune-associated genes in TCGA-GBM transcriptional data was used to filter prognosis-associated immune genes for unsupervised clustering analysis, and we investigated the immunological traits of distinct immune-associated clusters. A Glioblastoma Immunological Prognostic Score (GIPS) was developed to measure individual individuals’ immune characteristics. We then investigated the GIPS’s molecular and immunological properties, examined its ability to predict prognosis, and associated it with immunotherapy response and immunotherapy.

## RESULTS

### Identification of immune-related prognostic genes

**Figure 1:**
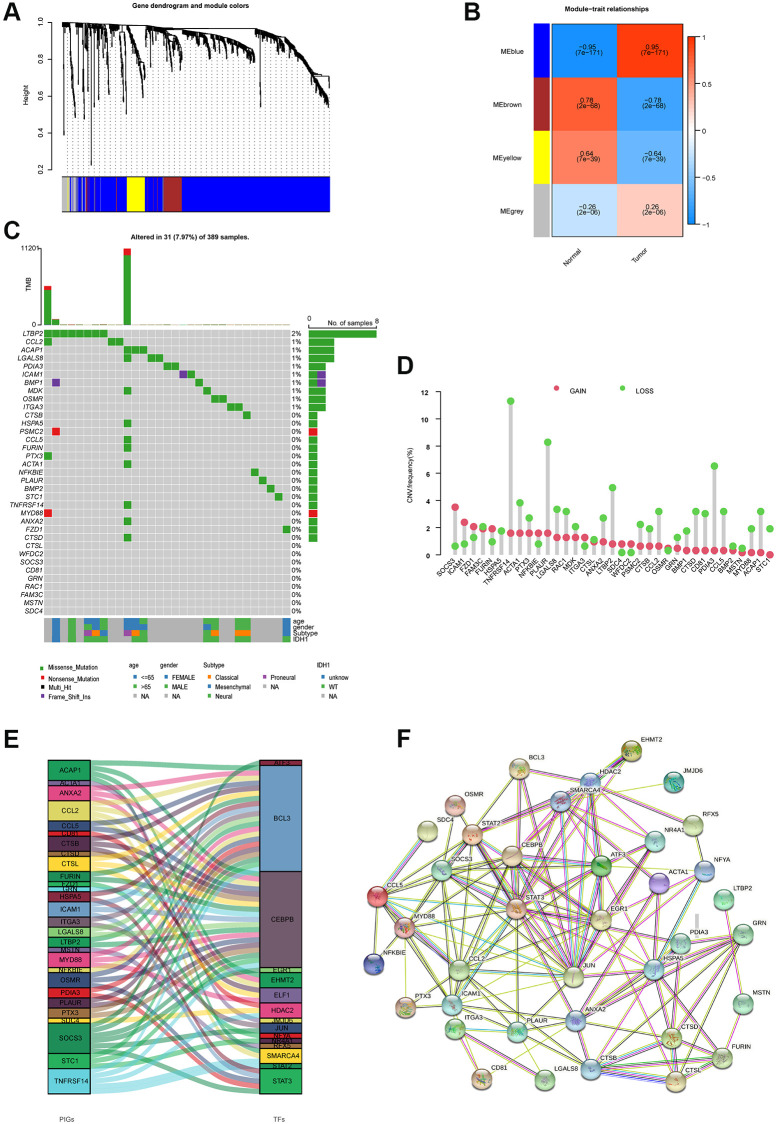
**Molecular characterizations of immune-related prognostic genes.** (**A**) Weighted gene coexpression network analysis (WGCNA) of immune-related differentially expressed genes with a soft threshold β = 7. (**B**) Gene modules related to HNSCC obtained by WGCNA. (**C**) Mutation frequency of 35 immune-related prognostic genes. (**D**) CNV variation frequencies of 35 immune-related prognostic genes in the TCGA-GBM cohort. (**E**) Transcription factors for 35 immune-related prognostic genes. (**F**) The network of the 35 immune-related prognostic genes.

The design of this study is shown in Supplementary Figure 1A. Differential analysis of immune-related genes from ImmPort and InnateDB yielded 802 differentially expressed immune-related genes. Supplementary Figure 1B and 1C show the results of the immune differential gene GO and KEGG pathway enrichment analyses. The candidate genes were subjected to WGCNA analysis in order to identify the immune-related center genes. Based on the scale-free network and the correlation coefficient of greater than 0.9, the soft-thresholding power was optimally set at (Supplementary Figure 1D and 1E). Four modules were identified based on the best soft threshold ability (Figure 1A and 1B). Among them, the blue module had the largest Pearson correlation coefficient and the smallest *P* value, so the genes in the blue module were selected for further analysis. The Univariate Cox analysis of the genes in the blue module revealed 35 prognostic-related immune genes with *p* ≤ 0.01 (Supplementary Figure 2). We then looked into the characteristics of the 35 genes in greater depth. These genes had a low mutation frequency but mostly copy number changes, with copy number loss dominating (Figure 1C and 1D). It's worth mentioning that the expression levels of most genes positively correlate with their copy number (Supplementary Figure 3). However, while CNV can explain many observed changes in prognostic-related immune genes expression, CNV is not the only factor involved in the regulation of mRNA expression. Furthermore, we identified BCL3 as the most common transcription factor for these prognostic immune genes and built a protein interaction network for them (Figure 1E and 1F).

### Two immune patterns of glioblastoma

Unsupervised consensus clustering of the expression of the above prognosis-related immune genes was used to investigate the immune pattern of glioblastoma. The empirical cumulative distribution function (CDF) plots show the consensus distributions for k (1–9) (Supplementary Figure 4A and 4B). Given the consensus matrix for the analysis, k = 2 appeared to be the best option. The consensus matrix demonstrates that an unsupervised algorithm based on these genes is capable of clearly distinguishing samples and that each sample in the cluster has a high degree of correlation (Supplementary Figure 4C–4E). As a result, we classified GBM patients into two groups, called immune clusters A and B, based on the expression of prognostically relevant immune genes. The two immune clusters had significantly different transcript expression profiles, according to principal component analysis (Supplementary Figure 4F).

**Figure 2:**
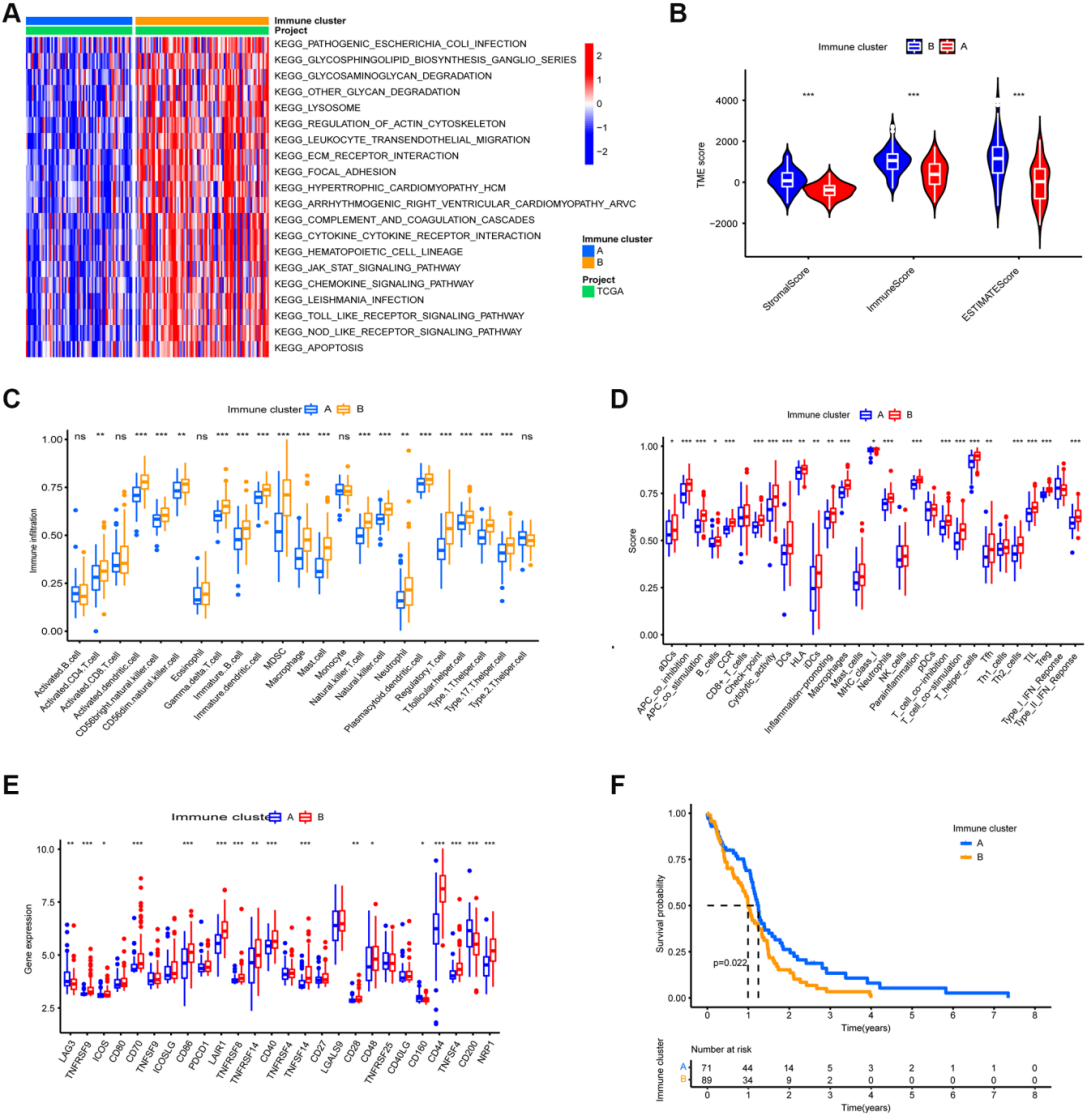
**TME Characterization of the two immune clusters.** (**A**) GSVA enrichment analysis of the two immune clusters. (**B**) TME scores of the two immune clusters. (**C**) Differential analysis of immune cell abundance between two immune clusters. (**D**) Differential of immune function between the two immune clusters. (**E**) Differential analysis of expression of immune checkpoints in the two m6A clusters. (**F**) Kaplan-Meier OS analysis in the two immune clusters. *P* = 0.022. (^*^, ^**^, and ^***^ indicate *p* ≤ 0.05, <0.01, and <0.001, respectively).

GSVA enrichment analysis revealed 83 differential pathways between the two immune clusters, and the top 20 KEGG pathways are shown in Figure 2A. Immune-related pathways such as the nod-like receptor signaling pathway, the chemokine signaling pathway, and leukocyte transendothelial migration were significantly enriched in immune cluster B, whereas DNA replication and nucleotide excision repair were significantly enriched in immune cluster A.

To reveal the immune landscape of the two immune clusters, we analyzed the immune microenvironmental features of the two immune clusters. Immune cluster B contained a higher number of both immune cells and stromal cells (Figure 2B). Further investigation revealed that almost all adaptive and innate immune cells were more infiltrated in immune cluster B, and immune cluster B's cellular function was more active (Figure 2C and 2D). Furthermore, the expression of immune checkpoints other than LAG3, CD160, and CD200 was more abundant in immune cluster B (Figure 2E). Because immune cluster B's stroma restricted immune cell entry into the tumor parenchyma and overexpressed immune checkpoints inhibited immune cell function, we classified immune cluster B as an immune tolerance phenotype. Immune cluster A had fewer immune cells and lower immune activity, indicating an immunodeficient phenotype. Furthermore, patients in immune cluster A had a better prognosis (Figure 2F).

### Construction of GIPS

**Figure 3:**
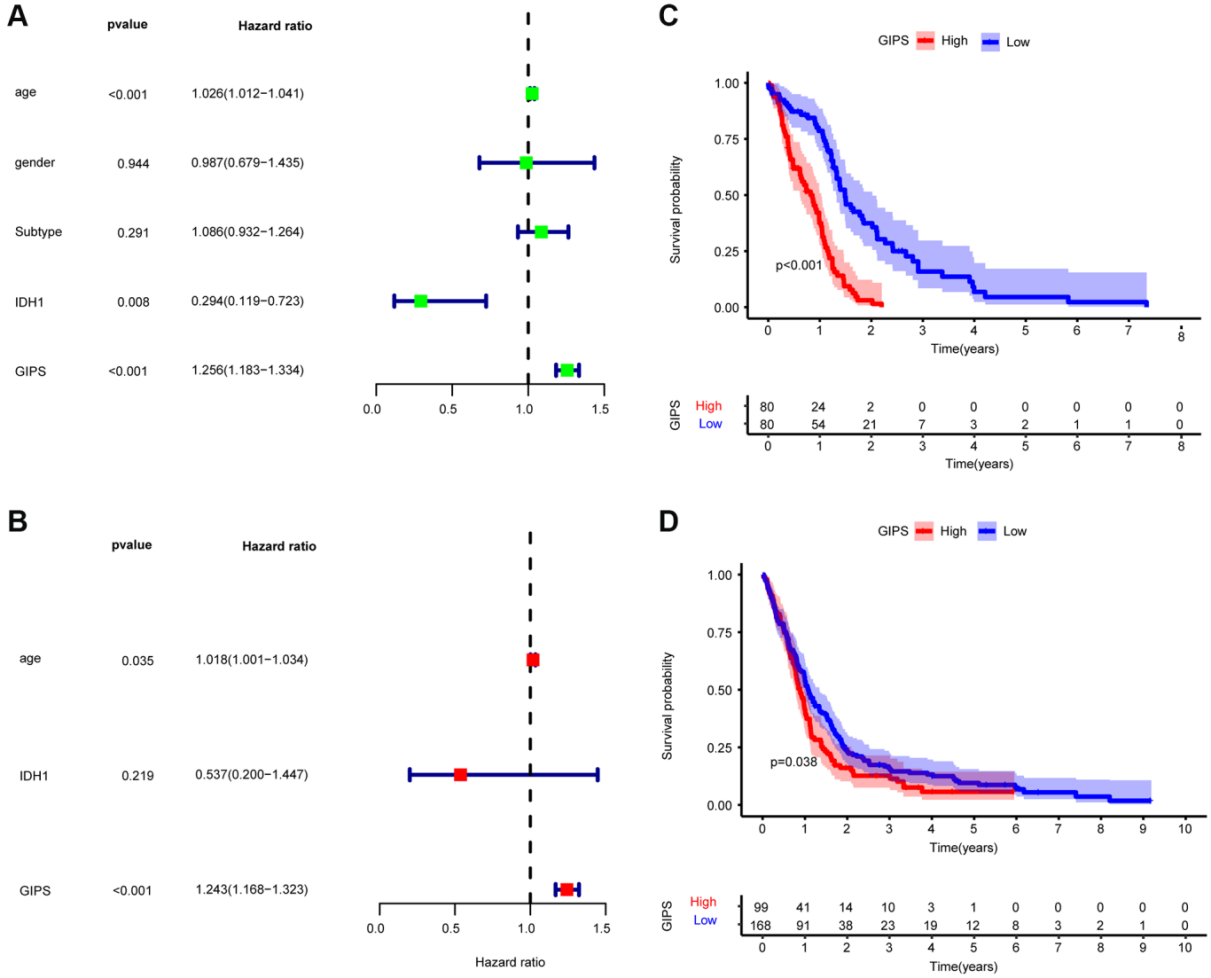
**Prognostic analysis of the GIPS subgroups.** (**A**) Univariate Cox analysis of clinical factors and the GIPS. (**B**) Multivariate Cox analysis of the factors significant in the univariate Cox analysis. (**C**) K-M analysis of the GIPS subgroups in TCGA-GBM cohort (*P* < 0.001). (**D**) K-M analysis of the GIPS subgroups in GEO cohort (*P* = 0.038).

Thirteen immune genes with independent prognosis and their coefficients were identified based on multivariate analysis of 35 prognosis-associated immune genes (Supplementary Table 1). We then constructed GIPS based on the coefficients for assessing the immune status, immunotherapy response, and prognosis of individual patients. Univariate Cox regression analysis showed that age, IDH1 status and GIPS were significantly associated with the prognosis of GBM (Figure 3A). Multifactorial Cox regression analysis confirmed that GIPS was an independent prognostic factor after adjusting for other factors (Figure 3B).

Taking the median GIPS as the cut-off value, low-GIPS patients had a better OS than high-GIPS patients (Figure 3C). Then, the role of GIPS was validated by using the GSE13041 dataset. As shown in Figure 3D, the patients in the low-GIPS subgroup had a significantly better prognosis than those in the high-GIPS subgroup, consistent with the results of the TCGA dataset.

### Molecular analysis of various GIPS subgroups

**Figure 4:**
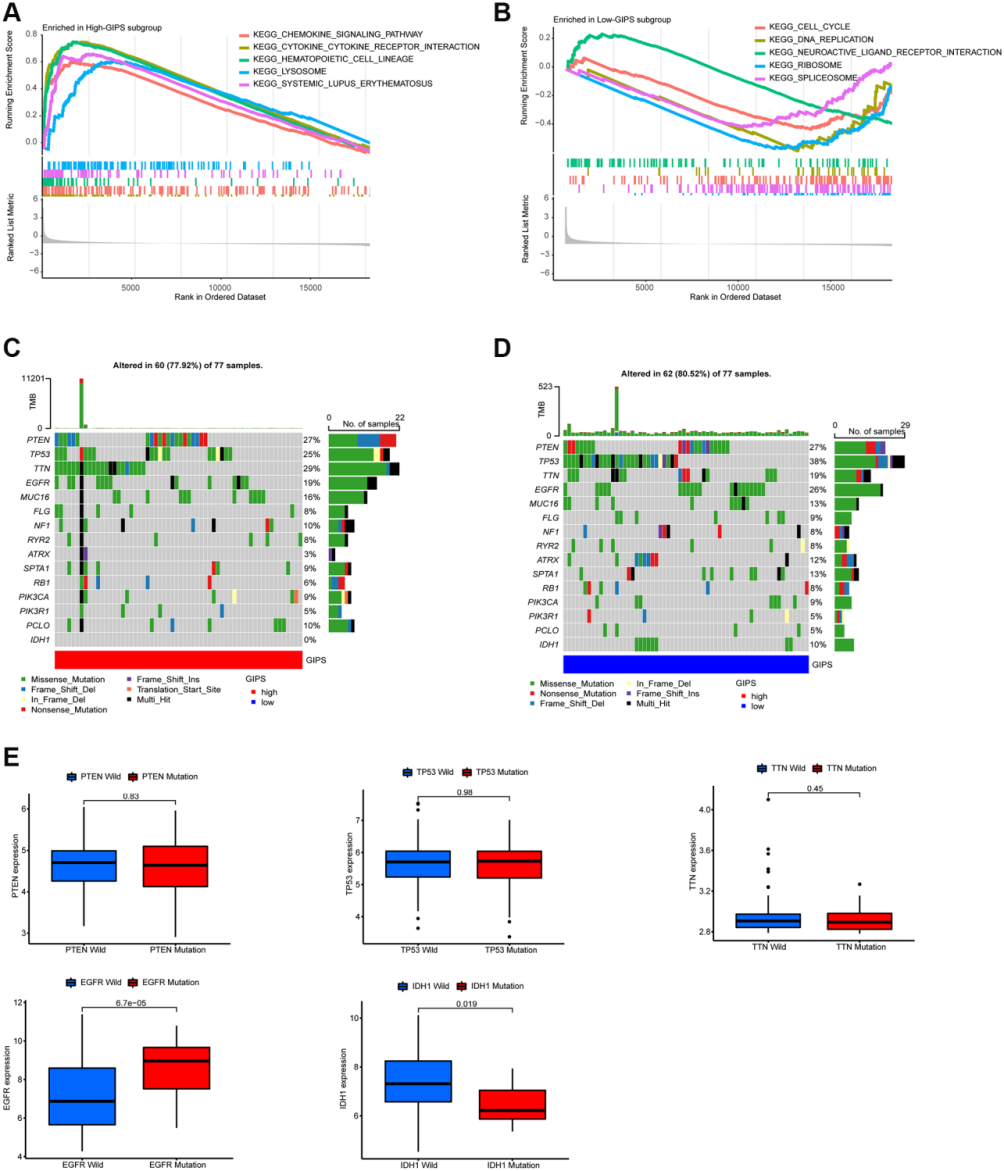
**Molecular traits of distinct GIPS subgroups.** (**A**) GSEA analysis in High-GIPS subgroup (*P* < 0.05). (**B**) GSEA analysis in Low-GIPS subgroup (*P* < 0.05). (**C**) Mutated genes (top 15) in High-GIPS subgroups. (**D**) Mutated genes (top 15) in Low-GIPS subgroups. (**E**) The correlation between expression level and mutations of genes (PTEN, TP53, TTN, IDH1 and EGFR).

GSEA was used to identify the set of genes that were enriched in various GIPS subgroups. The genomes of High-GIPS patients were enriched in cytokine-cytokine receptor interaction and the chemokine signaling pathway, whereas the genomes of Low-GIPS patients were enriched in the cell cycle and DNA replication (Figure 4A and 4B).

We then examined the gene mutations to gain a better understanding of the immunological nature of the GIPS subgroup from a biological standpoint. Figure 4C and 4D depict the top 15 genes in the GIPS subgroups with the highest mutation rates. The most common type of mutation was missense mutation, followed by frameshift deletion and nonsense mutation. PTEN, TP53, TTN, and EGF2 mutation rates were greater than 15% in both groups. PTEN, TP53, and TTN expression levels were not associated with mutations, whereas IDH1 mutations were associated with decreased expression and EGFR mutations with increased expression (Figure 4E). Notably, the mutation rate of IDH1 was 0% in the high GIPS subgroup and 10% in the low GIPS subgroup.

### Immunological characteristics of various GIPS subgroups

**Figure 5:**
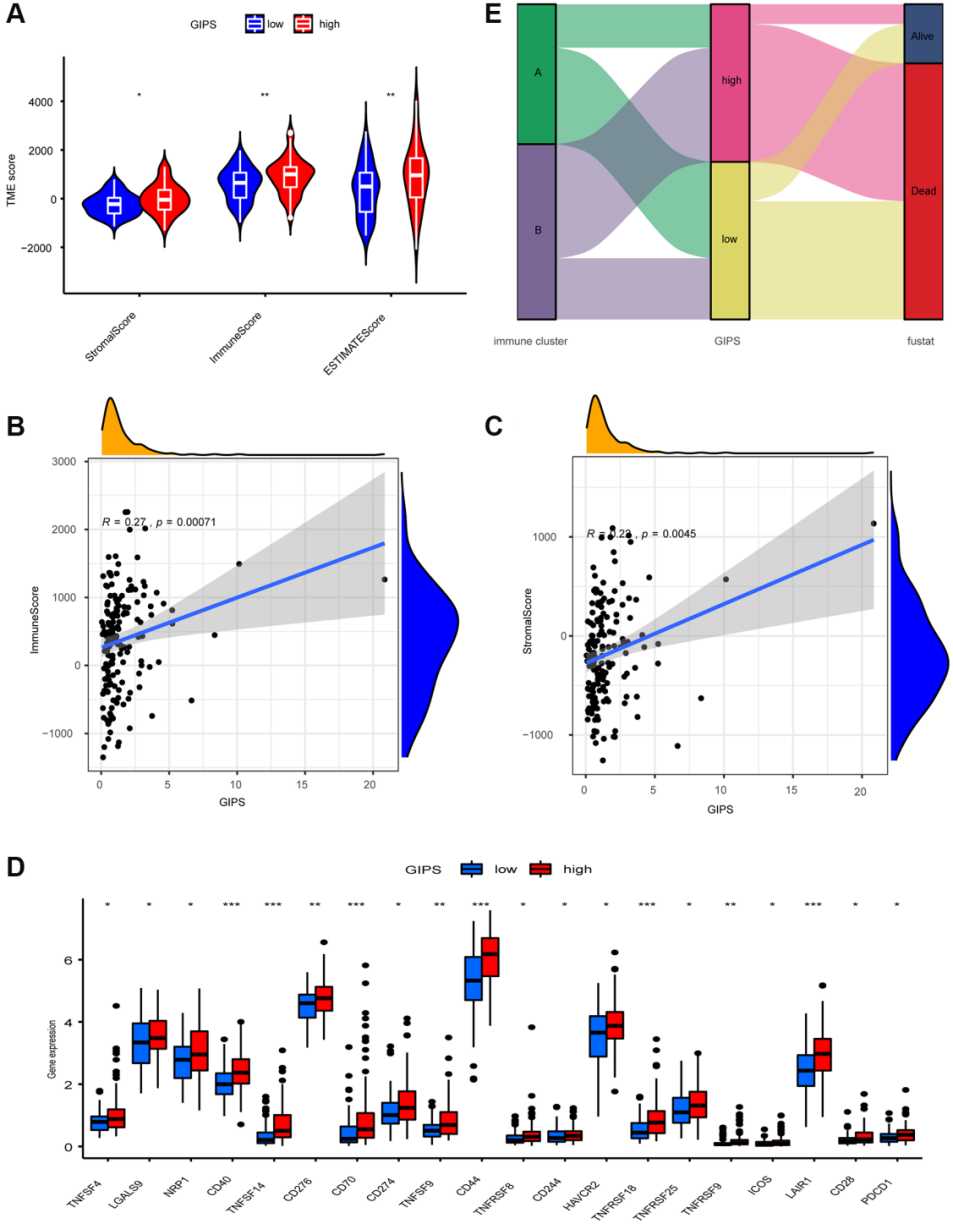
**TME Characterization of distinct GIPS subgroups.** (**A**) TME scores of the distinct GIPS subgroups. (**B**) Spearman correlation analysis of GIPS scores with immune scores. R = 0.27, *P* ≤ 0.001. (**C**) Spearman correlation analysis of GIPS scores with stromal scores. R = 0.22, *P* ≤ 0.01. (**D**) Differential analysis of expression of immune checkpoints in the different GIPS subgroups. (**E**) Alluvial diagram of GBM patient immune cluster and GIPS.

The High-GIPS subgroup had higher immune and stroma scores (Figure 5A), and correlation analysis between GIPS and TME revealed that GIPS was significantly positively correlated with immune (R = 0.27, *p* = 2.2e-16) and stroma (R = 0.22, *p* = 0.0045, Figure 5B and 5C) scores. There were more innate and adaptive immune cells, EMT and Pan-F-TBRS in the High-GIPS subgroup, while there was more DNA damage repair, DNA replication and mismatch repair in Low-GIPS subgroup (Supplementary Figure 5A and 5B). Moreover, almost all common immune checkpoints had higher expression in the High-GIPS subgroup (Figure 5D). According to the alluvial diagram, the majority of low GIPS patients belong to immune cluster A (immune tolerance pattern), while the majority of high GIPS patients belong to immune cluster B (immune deficiency pattern) (Figure 5E).

### Immunotherapy response in various GIPS subgroups

**Figure 6:**
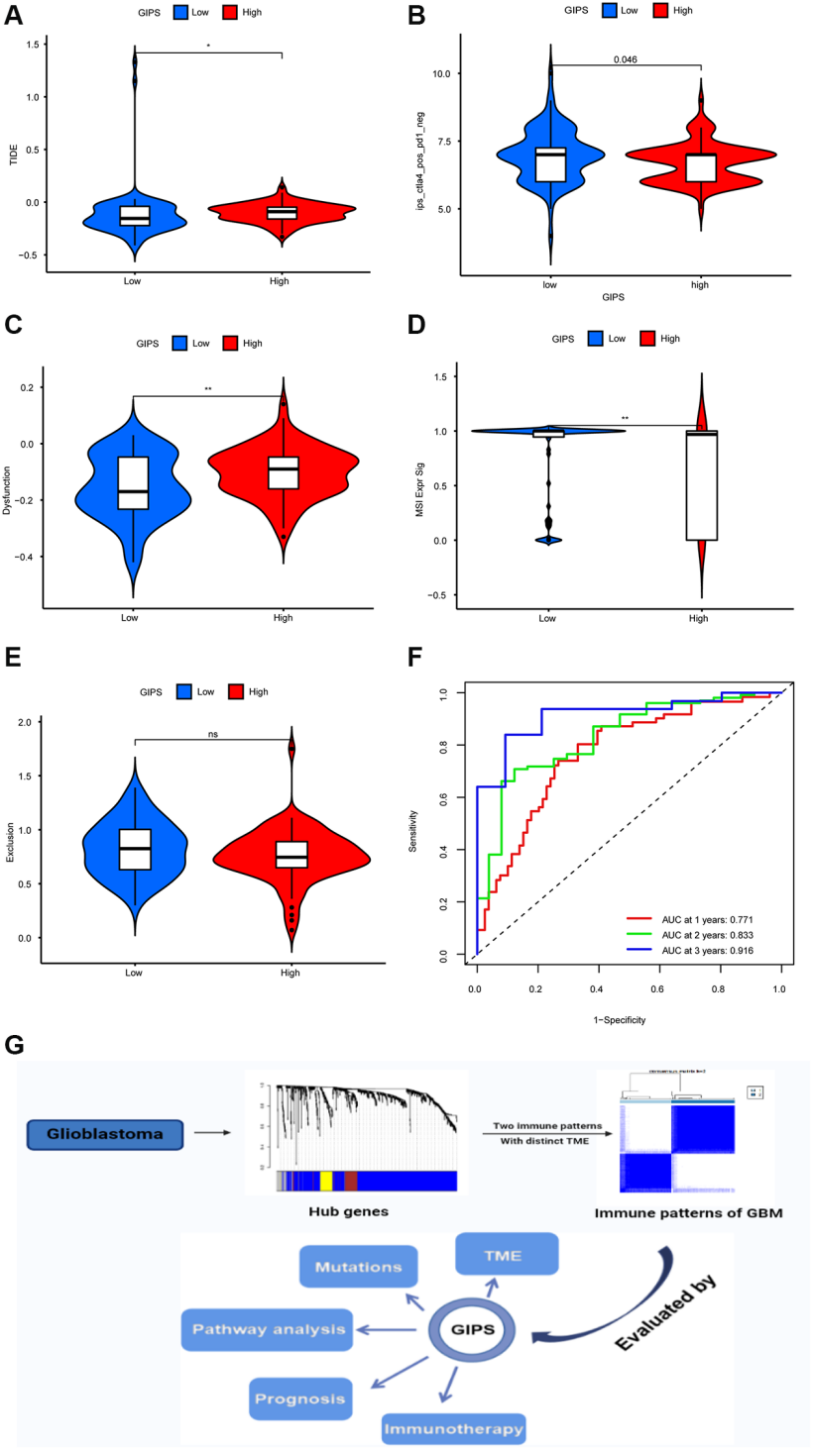
**Immunotherapy response in different GIPS subgroups.** (**A**) Differential analysis of TIDE score between GIPS subgroups. (**B**) Effectiveness of immunotherapy in GIPS subgroups. (**C**) Differential analysis of dysfunction score between GIPS subgroups. (**D**) Differential analysis of MSI score between GIPS subgroups. (**E**) Differential analysis of T-cell exclusion score between GIPS subgroups. (**F**) ROC curves of GIPS for predicting 1-, 2-, and 3-year survival in TCGA GBM cohorts. (**G**) Graphical summary of this study.

TIDE was used to assess immunotherapy response in various GIPS subgroups. Patients with high TIDE scores have a lower immunotherapy response, indicating that immunotherapy is less likely to benefit them. TIDE scores were higher in the High-GIPS subgroup than in the Low-GIPS subgroup, indicating that ICI therapy may benefit Low-GIPS patients more (Figure 6A). The effects of anti-CTLA4 therapy on different GIPS subgroups were then compared. The Low-GIPS subgroup did fare better in terms of treatment outcomes (Figure 6B).

To determine the reasons for the differences in immunotherapy response, we compared microsatellite instability (MSI), T-cell exclusion, and T-cell dysfunction scores in the two subgroups. Low-GIPS subgroup had a higher microsatellite instability (MSI) score, while the High-GIPS subgroup had a higher T-cell dysfunction score, but there was no difference in T-cell exclusion between the 2 subgroups (Figure 6C–6E). Furthermore, ROC curve analysis showed that the AUC values of GIPS reached 0.771 at 1 year, 0.833 at 2 years, and 0.916 at 3 years (Figure 6F) and were higher than the AUC values of T-cell inflammatory signature (TIS) and TIDE (Supplementary Figure 5C–5E).

## DISCUSSION

Glioblastoma is a type of central nervous system tumor that develops from glial stem cells [7]. Despite a variety of treatments, the prognosis for patients is frequently extremely poor [8]. Immunotherapy appears to offer a ray of hope for patients with glioblastoma. However, many patients do not respond well to immunotherapy and not only benefit less but also suffer from a slew of side effects [9]. According to some studies, the tumor microenvironment influences immunotherapy responses [10, 11]. Furthermore, there are no validated biomarkers to predict immunotherapy response and overall survival. As a result, in this study, we attempted to analyze the tumor microenvironment of glioblastoma using big data in order to elucidate the causes affecting immunotherapy response in glioblastoma patients and to propose corresponding treatment plans based on glioblastoma immune microenvironment characteristics.

Based on the GBM immune gene dataset, we identified 35 immune-related prognostic genes using WGCNA and K-M analysis in this study. Although the mutation rate was low, most of these genes had copy number loss. It has been demonstrated that gene copy number loss is linked to tumorigenesis [12, 13]; for example, the loss of ErbB4 receptor tyrosine kinase is linked to the development of glioblastoma [14]. Thus, immune gene copy number loss may play a role in the development of glioblastoma. Based on an unsupervised clustering analysis of these 35 genes, we discovered that glioblastoma has two immune patterns. Immune cluster A is primarily enriched in DNA replication and nucleic acid repair pathways, with less immune cell infiltration and low immune function, defining the immunodeficiency pattern. Immune cluster B had a high infiltration of immune cells and active immune function and was primarily enriched with immune-related pathways. As a result, in immunodeficiency pattern patients, improving immune function may be beneficial in terms of prognosis. Numerous studies have found that a dense infiltration of T cells, particularly cytotoxic [15, 16], predicts a favorable outcome. However, immune cluster B did not show the expected survival advantage. Further research revealed that increased stromal cells in immune cluster B limited the function of immune cells infiltrating the tumor parenchyma, while highly expressed immune checkpoints and increased Treg cells inhibited T cells' tumor killing function. As a result, immune cluster B displayed an immune tolerance pattern. Furthermore, soluble factors secreted by stromal cells can induce microenvironment-mediated drug resistance [17]. As a result, rational drug combinations capable of targeting both tumor cells and the microenvironment may be the key to overcoming therapeutic resistance [18, 19].

GIPS was created using multivariate cox analysis to assess the immune pattern, immunotherapy response, and prognosis of individual patients. When compared to the High-GIPS subgroup, the Low-GIPS subgroup had a higher survival rate and a longer survival time. The validity of the model was subsequently demonstrated by a validation cohort. We then investigated mutations in distinct GIPS subgroups to better understand the immunological landscape of these subgroups. As reported earlier, missense mutation, followed by frameshift deletion and nonsense mutation. Notably, the mutation rate of IDH1 reached 10% in the Low-GIPS subgroup while there were no mutations in the High-GIPS subgroup. According to some studies, the IDH1 mutation is an independent predictor of longer overall survival (OS) and progression-free survival (PFS) in GBM patients [20, 21]. The FAT1-ROS-HIF-1 signaling pathway is activated by the IDH1 mutation, which suppresses malignant glioma [22]. Thus, Low-GIPS patients with high IDH1 mutations have a better prognosis than High-GIPS patients who do not have IDH1 mutations, which is consistent with our survival findings.

Understanding the TME can aid in the discovery of new ways to treat GBM, as well as altering the TME to improve the efficacy of immunotherapy. Tumor microenvironments differ between the two GIPS subgroups. Multiple immune cells, such as natural killer cells, macrophages, and Treg cells, are more abundant in the High-GIPS subgroup. Tumor-associated macrophages contribute to patients' poor prognosis by promoting glioblastoma growth and angiogenesis [23]. Moreover, a large infiltration of Treg cells leads to immunosuppression [24]. As a result, a number of studies have proposed therapeutic approaches that target tumor-associated macrophages [23]. For example, promoting macrophage polarization in combination with immune check inhibitors aids in the treatment of glioblastoma [25]; and blocking macrophage-associated immunosuppression to regulate glioblastoma angiogenesis [26]. These approaches, however, need to be investigated further. The Low-GIPS subgroup has less immune cell infiltration but a greater ability to repair damage.

TIDE was then used to assess the immunotherapy responsiveness of different GIPS subgroups. Patients in the High-GIPS subgroup had higher immune cell infiltration and TIDE and T cell dysfunction scores than those in the Low-GIPS subgroup, suggesting that their lower immunotherapy response could be due to immune evasion caused by T cell dysfunction. The Low-GIPS subgroup, on the other hand, had higher MSI scores and lower TIDE scores than the High-GIPS subgroup, indicating that these patients had less immune evasion and more MSI. The high mutational load caused by MSI has been shown to make the tumor immunogenic and sensitive to immune checkpoint inhibitors. We directly compared the responses of different GIPS subgroups to anti-CTLA4 therapy to further validate GIPS's ability to predict patients’ responses to immunotherapy. Patients in the Low-GIPS subgroup were found to be indeed more sensitive to immunotherapy.

The TIDE score is an algorithm that simulates tumor immune evasion mechanisms [27] and can be used to assess immunotherapy response in patients with a wide range of tumors, including bladder cancer, lung adenocarcinoma, and melanoma [28, 29]. Furthermore, the Tumor Inflammation Signature (TIS) provides quantitative and qualitative information about TME, which has been shown in a pan-cancer cohort to correlate with benefit from anti-PD -1 therapy [30]. Nevertheless, TIDE and TIS are both concerned with assessing the function and condition of T cells and do not truly reflect the impact of the tumor microenvironment on immunotherapeutic responses [2, 31], such as the important role that tumor-associated macrophages play in glioblastoma. Furthermore, both signatures are concerned with the patient's response to immunotherapy rather than the patient's overall survival, and life expectancy is an important consideration when making a clinical decision. GIPS has a higher predictive value than TIDE and TIS, and it may be a more valuable predictor for assessing a patient's immune condition, immunotherapy response, and prognosis.

In conclusion, glioblastoma has two immune modalities, immune tolerance and immunodeficiency, with distinct immune microenvironments, tumor-associated macrophages being one of the most promising new therapeutic targets. GIPS is a promising biomarker for assessing immune evasion mechanisms, immunotherapy responses, and prognosis in patients, but more research is needed to confirm its utility (Figure 6G).

## METHODS

### Data collection and processing

The TCGA-GBM dataset and GSE13041 were used to obtain GBM transcriptome data. GTEx was used to obtain RNAseq transcriptome data for healthy human tissues. The GTEx and TCGA datasets were combined and reconciled using quantile normalization and batch effect removal using svaseq. Immune-related gene lists were obtained from the databases ImmPort and InnateDB.

### Identification of immune-related prognostic genes

Differential analysis of immune-related genes from ImmPort and InnateDB yielded 802 differentially expressed immune-related genes. Then, WGCNA was performed to identify these genes. Based on the scale-free network and the correlation coefficient of greater than 0.9, the soft-thresholding power was optimally set at 7. Univariate Cox analysis of the genes in the blue module revealed 35 prognostic-related immune genes (*p* ≤ 0.01).

### Unsupervised consensus clustering based on immune-related prognostic genes

According to expression levels of the 35 prognostic-related immune genes, unsupervised clustering analysis was used for identification of various immune patterns and patient classification for further analyses. Cluster numbers and their stabilities were evaluated using a consistent clustering algorithm.

### Gene set variation analysis

Gene set “c2.cp.kegg.v6.2.symbols” was obtained from the MSigDB and GSVA enrichment analysis used to identify different immune patterns using “GSVA” package on R, with adjusted *p* ≤ 0.05 indicating statistical significance. In non-parametric and unsupervised approaches, GSVA is used for estimation of pathway and biological process changes in gene expression datasets.

### Estimation of TME immune trait

The ssGSEA enrichment fraction was used to calculate the relative abundances of each TME-infiltrating cell per sample. Charoentong's study, which annotated human immune cell subtypes, immune checkpoints, and EMT, provided the genomes used to mark each TME-infiltrating immune cell type.

### Establishment and subsequent validation of the GIPS

Among 35 immune-related prognostic genes, multifactorial Cox regression analysis was used to identify genes with significant effects on OS. The GIPS for each sample was calculated by multiplying the expression values of specific genes by their Cox model weights and then summing them. Log-rank tests were used to assess GIPS's prognostic ability in the TCGA and GEO cohorts using Kaplan-Meier (K-M) survival curves. GIPS’s independent prognostic value was validated using univariate and multivariate Cox regression analyses.

### Statistical analysis

The limma R package was used to analyze differential gene expression. The statistical difference between the two groups was calculated using the Wilcoxon rank sum test. For comparisons of more than two groups, the Kruskal-Wallis test was used. For all statistical analyses, R software was used.

### Ethics approval and consent to participate

Patient information is available in public databases that was collected with patients’ informed consent.

### Consent for publication

All authors give consent for the publication of this manuscript in Aging -us.

### Availability of data and materials

The data used in the study are described in detail in “Data collection and processing”.

## Supplementary Materials

Supplementary Figures

Supplementary Table 1
