# Supplementary material for: Glioblastoma: two immune subtypes under the surface of the cold tumor
Source: Aging (Albany NY). 2022 May 23;14(10):4357–75. doi: 10.18632/aging.204067 (PMC9186767; doi:10.18632/aging.204067)
Supplement: Supplementary Figures [file aging-14-204067-s001.pdf]

SUPPLEMENTARY FIGURES

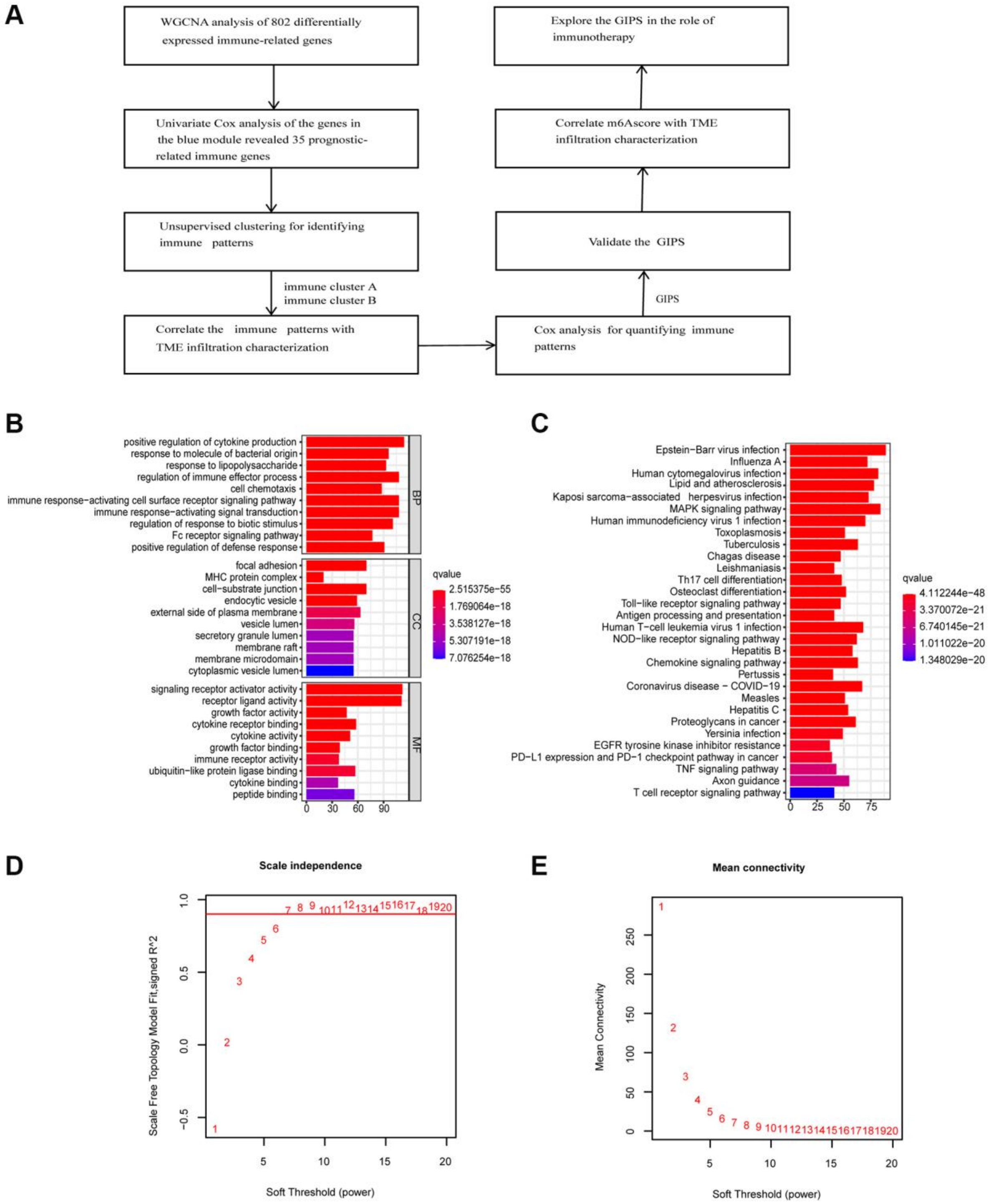

Supplementary Figure 1. Differentially expressed immune-related genes in GBM. (**A**) This study's design overview. (**B**) GO enrichment analysis of the immune-related DEGs. (**C**) KEGG enrichment analysis of the immune-related DEGs. (**D**) The horizontal line denotes a threshold value of 0.9. (**E**) WGCNA's optimal soft threshold is 7.

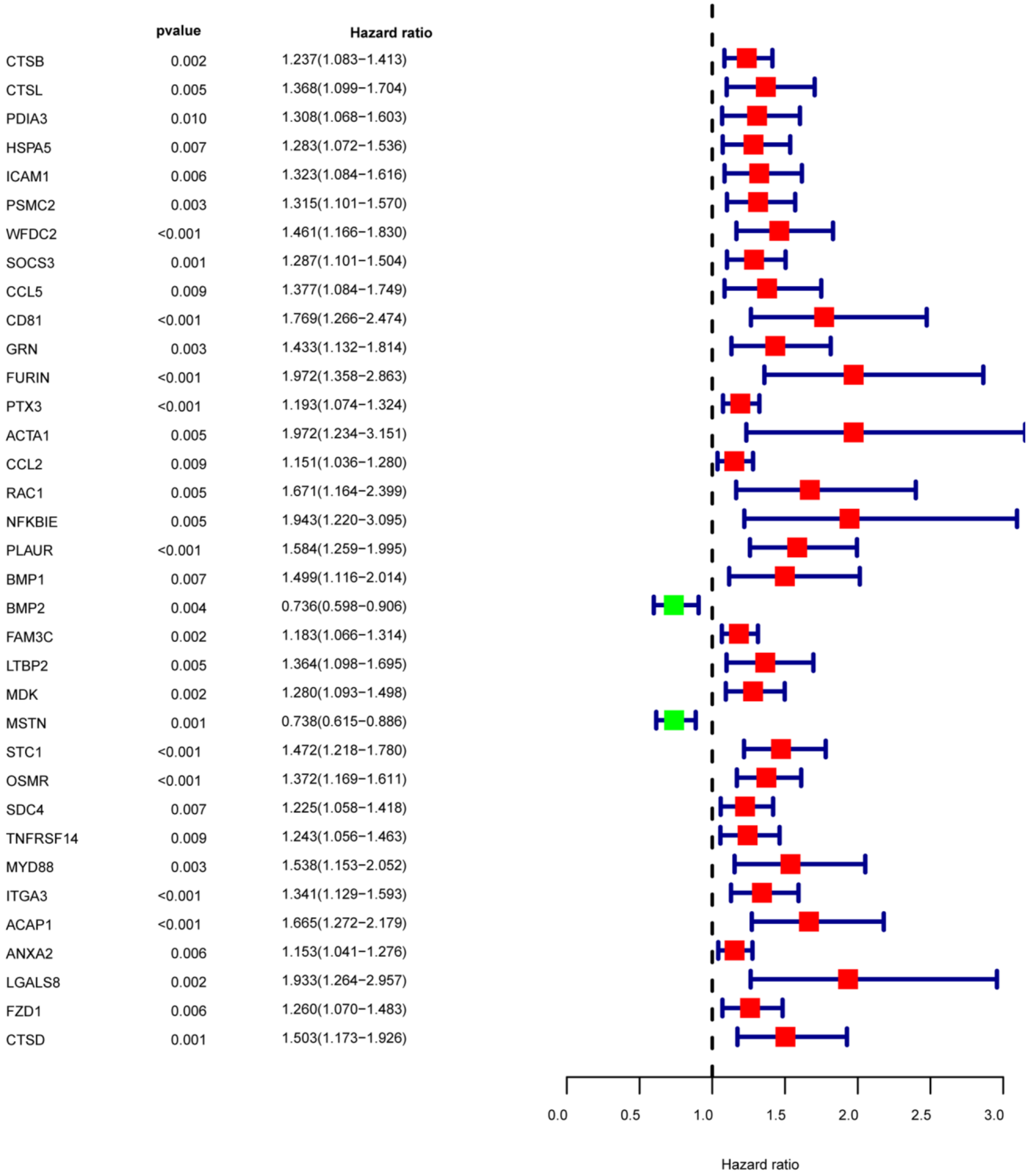

Supplementary Figure 2. Univariate Cox analysis of the genes in the blue module. Forest plot of 35 immune-related prognostic genes.

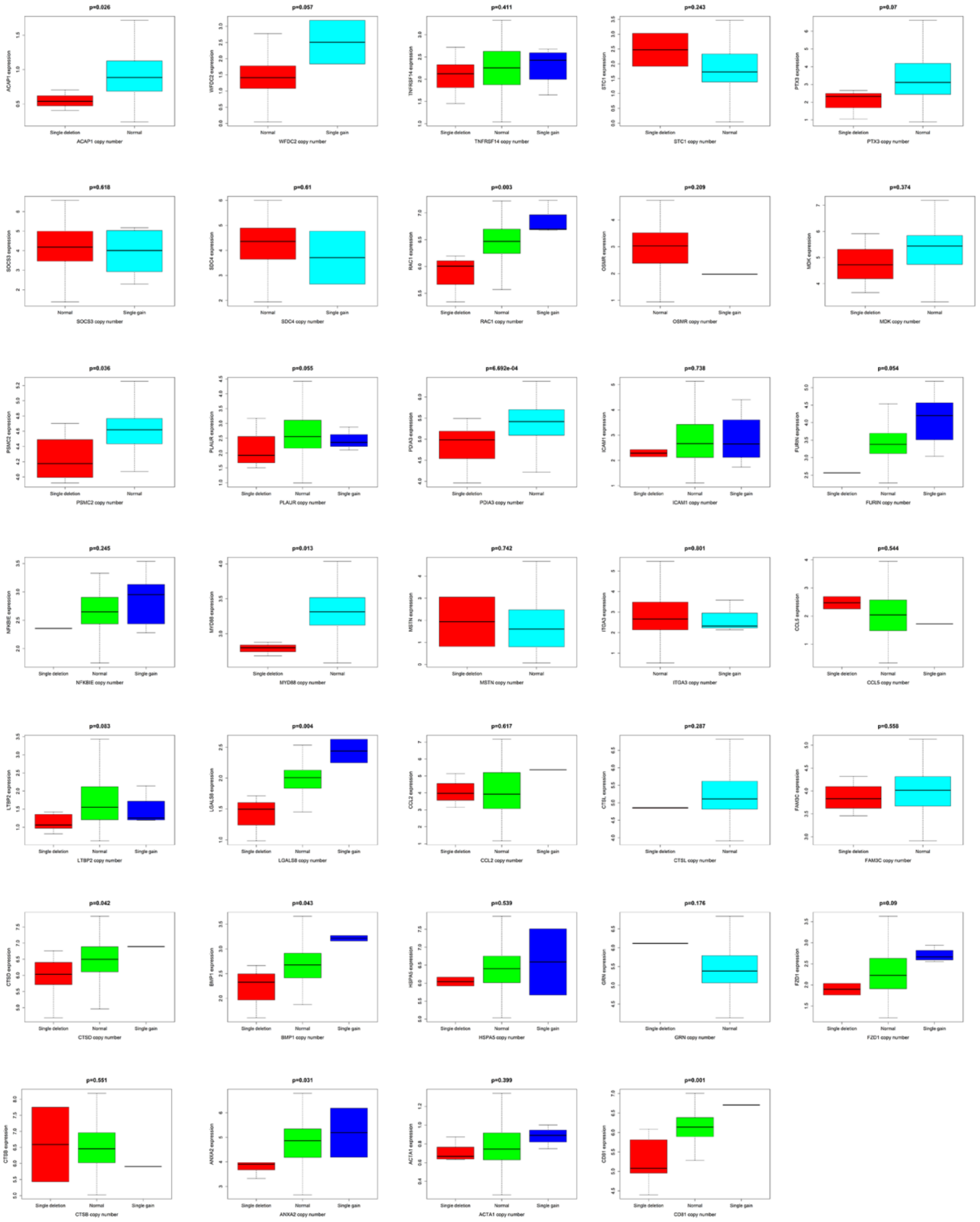

Supplementary Figure 3. The correlation between expression level and copy number of the 35 prognostic-related immune genes.

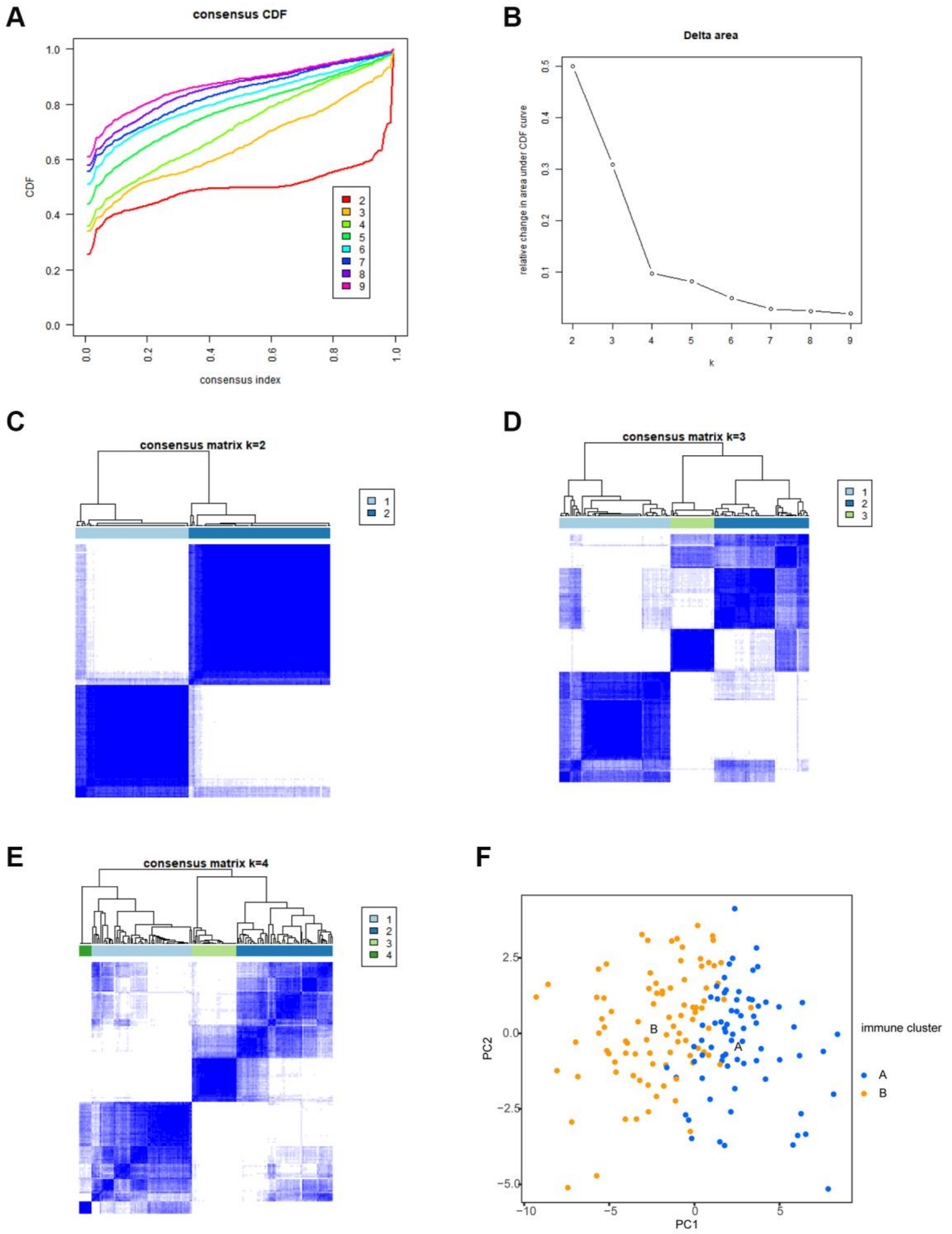

Supplementary Figure 4. Unsupervised clustering analysis in GBM meta cohort. (**A** and **B**) The empirical cumulative distribution function (CDF) plots show the consensus distributions for k (1–9). (**C**–**E**) Consensus matrix of TCGA-GBM dataset (k = 2–4). (**F**) Principal component analysis of expression profiles in the two immune clusters.

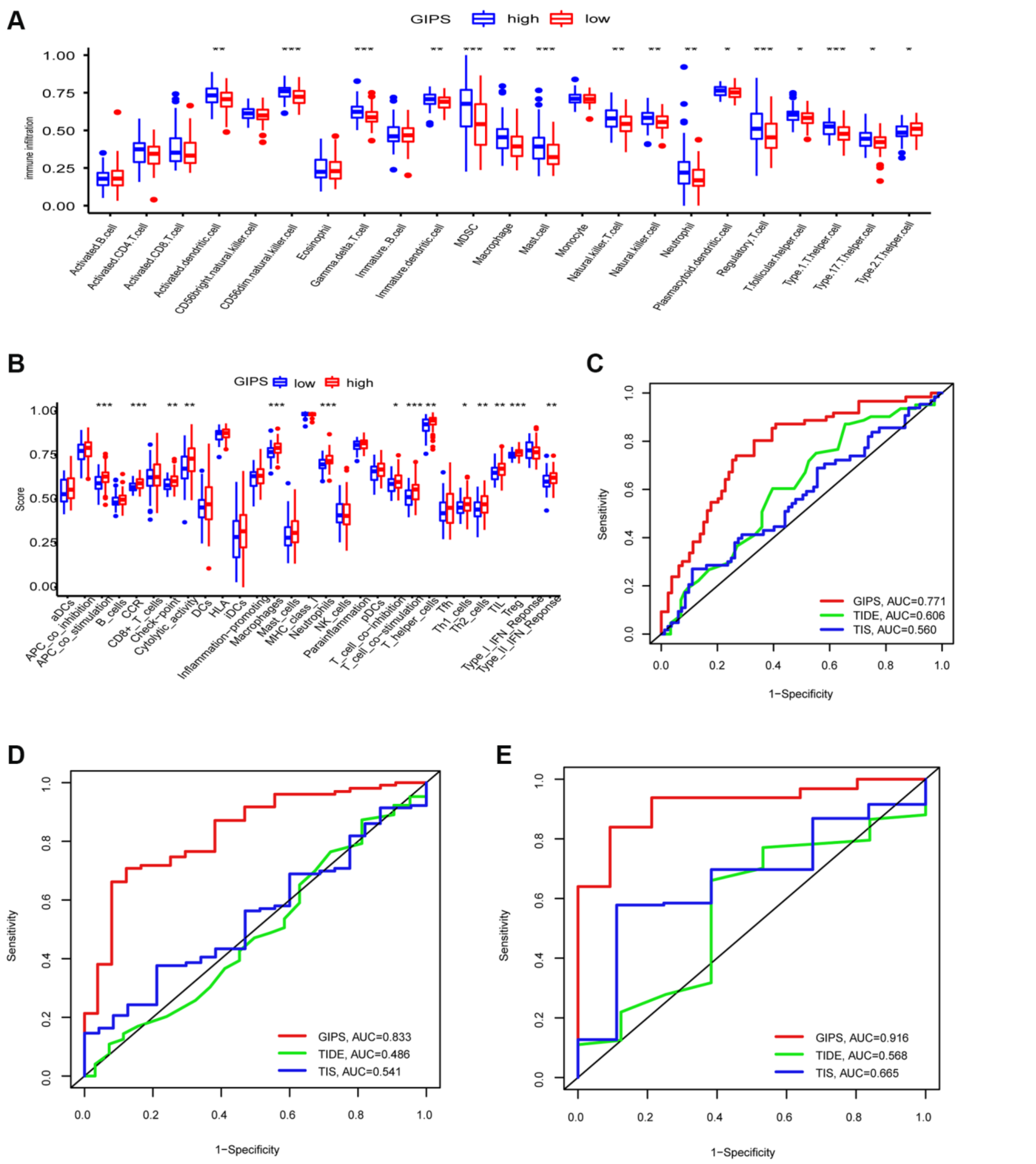

Supplementary Figure 5. The immune cells infiltration and immune-related function of distinct GIPS subgroup. (**A**) Differential analysis of immune cell abundance between two GIPS subgroups. (**B**) Differential of immune function between the two GIPS subgroups. (**C**–**E**) ROC analysis of GIPS, TIS, and TIDE on OS at 1-, 2-, and 3-year.
