# Supplementary material for: Glioblastoma: two immune subtypes under the surface of the cold tumor
Source: Aging (Albany NY). 2022 May 23;14(10):4357–75. doi: 10.18632/aging.204067 (PMC9186767; doi:10.18632/aging.204067)
Supplement: Supplementary Table 1 [file aging-14-204067-s002.pdf]

SUPPLEMENTARY TABLE

Supplementary Table 1. Coefficients of 13 immune genes.

Gene	coef
ICAM1	−0.313685717
PSMC2	0.255439674
WFDC2	0.34938006
CD81	0.614962106
ACTA1	0.813674408
PLAUR	0.283402033
FAM3C	0.120266822
MSTN	−0.31394654
OSMR	0.273880225
TNFRSF14	−0.178961902
ITGA3	0.200600623
ACAP1	0.803335701
ANXA2	−0.287133365
